# Emotion Recognition from Skeletal Movements

**DOI:** 10.3390/e21070646

**Published:** 2019-06-29

**Authors:** Tomasz Sapiński, Dorota Kamińska, Adam Pelikant, Gholamreza Anbarjafari

**Affiliations:** 1 Institute of Mechatronics and Information Systems Lodz University of Technology, 90-924 Lodz, Poland; 2 iCV Lab, Institute of Technology, University of Tartu, 51014 Tartu, Estonia; 3 Faculty of Engineering, Hasan Kalyoncu University, 27000 Sahinbey, Gaziantep, Turkey; 4 Institute of Digital Technologies, Loughborough University London, London E15 2GZ, UK

**Keywords:** emotion recognition, gestures, body movements, Kinect sensor, neural networks, deep learning

## Abstract

Automatic emotion recognition has become an important trend in many artificial intelligence (AI) based applications and has been widely explored in recent years. Most research in the area of automated emotion recognition is based on facial expressions or speech signals. Although the influence of the emotional state on body movements is undeniable, this source of expression is still underestimated in automatic analysis. In this paper, we propose a novel method to recognise seven basic emotional states—namely, happy, sad, surprise, fear, anger, disgust and neutral—utilising body movement. We analyse motion capture data under seven basic emotional states recorded by professional actor/actresses using Microsoft Kinect v2 sensor. We propose a new representation of affective movements, based on sequences of body joints. The proposed algorithm creates a sequential model of affective movement based on low level features inferred from the spacial location and the orientation of joints within the tracked skeleton. In the experimental results, different deep neural networks were employed and compared to recognise the emotional state of the acquired motion sequences. The experimental results conducted in this work show the feasibility of automatic emotion recognition from sequences of body gestures, which can serve as an additional source of information in multimodal emotion recognition.

## 1. Introduction

People express their feelings through different modalities. There is evidence that the affective state of individuals is strongly correlated with facial expressions [1], body language [2] voice [3] and different types of physiological changes [4]. On the basis of external behaviour one can easily determine the internal state of the interlocutor. For example, burst of laughter generally signals amusement, frowning signals nervousness or irritation, crying is closely related to sadness and weakness [5,6,7]. Mehrabian formulated the principle 7-38-55, according to which the percentage distribution of the message is as follows: 7% verbal signals and words, 38% strength, height, and rhythm and 55% body movements and facial expressions [8]. This suggests that words serve in particular to convey the information and the body language to form conversation or even to substitute the verbal communication. However, it has to be emphasised that this relation is applicable only if a communicator is talking about their feelings or attitudes [9].

Currently, human-computer interaction (HCI) is one of the most rapidly growing fields of research. The main goal of HCI is to facilitate the interaction using several parallel channels of communication between the user and the machine. Although computers are now a part of human life, the relation between human and computer is not natural. Knowledge of the emotional state of the user would allow the machine to boost the effectiveness of cooperation. That is why affect detection became an important trend in pattern recognition and has been widely explored, especially in the case of facial expressions and speech signals [10]. Body gestures and posture receive considerably less focus. With recent developments and the increasing reliability of motion capture technologies, the literature about automatic recognition of expressive movements has been increasing in quantity and quality. Despite the rising interest in this topic, affective body movements in automatic analysis are still underestimated [11].

The most natural and intuitive method for body movement projection is based on the skeleton, which represents hierarchically arranged joint kinematics along with body segments [12]. In the past, research on body tracking was based on video data, which made it extremely challenging and usually amounted to single frame analysis [13,14,15]. However, the definition of motion is a change in position over time, thus it should be described as a set of consecutive frame sequences. Skeleton tracking has become much easier with the appearance of motion capture systems, which automatically generate the human skeleton represented by 3-dimensional (3D) coordinates. Additionally, it brought up an increase of research on body movement, such as unusual event detection and crime prevention [16,17,18,19,20].

Affective movement may be described by displacement, distance, velocity, acceleration, time and speed by extracting dynamic features from analysed model. For example in Reference [21], the authors were tracking trajectories of head and hands from a frontal and a lateral view. They combined shape and dynamic expressive gesture features, creating a 4D model of emotion expression that effectively classified emotions according to their valence and arousal. Dynamic features were also considered in Reference [22], where the authors suggested that the timing of the motion is an accurate representation of the properties of emotional expressions.

Very promising results are presented in Reference [23]. The authors analysed Microsoft Kinect v2. recordings of body movements expressing five basic emotions, namely, anger, happiness, fear, sadness and surprise. They used a deep neural network consisting of stacked RBMs, which outperformed all other classifiers, achieving an overall recognition rate of 93%. However, it must be emphasised that the superior performance is associated with the type of analysed data. In Reference [23] emotions are represented as predetermined gestures (each emotion is assigned to particular type of gesture, for example, power pose to happiness). The actors/actresses are instructed how to present particular emotional state prior to recording. Such an approach narrows the research down to the posture recognition problem, which may not be as effective with more complex gestures, despite such promising results.

More viable research is presented by Kleinsmith et al. in Reference [24], where the Gypsy 5 motion capture system was used to record the spontaneous body gestures of Nintendo Wii sports games players. The authors used low-level posture configuration features to create affective movement models for states of concentration, defeat and triumph. An overall accuracy of 66.7% was obtained using a multilayer perceptron. The emotional behaviour of Nintendo Wii tennis players was also analysed in Reference [25]. The authors based their experiment on time-related features such as body segment rotation, angular velocity, angular frequency, orientation, angular acceleration, body directionality and amount of movement. Results obtained using recurrent neural network (RNN), whose average recognition rate is 58.4%, are comparable to human observers’ benchmarks.

More recent research [26] presents analysis of human gait recordings performed by professional actors/actresses, captured by Vicon system. The motion data is encoded with HMMs, which are subsequently used to derive a Fisher Score (FS) representation. SVM classification is performed in the HMM-based FS space. The authors obtained a total average recognition rate of 78% for the same subject and 69% for interpersonal recognition. Classification was performed for four emotional states: neutral, joy, anger and sadness. In Reference [27], Vicon was used to collect a full body dataset of emotion including anger, happiness, fear and sadness, expressed by 13 subjects. The authors proposed a stochastic model of the affective movement dynamics using hidden Markov models, performance of which was tested with SVM classifier and resulted in 74% recognition rate.

Despite much lower accuracy compared to affective speech or facial expressions, gesture analysis can serve as a complement to a multimodal system. For example in Reference [28], the authors expanded their studies on emotional facial expressions by analysing sequences of images presenting the motion of arms and upper body. They used a deep neural networks model to recognise dynamic gestures with minimal image pre-processing. By summing up all the absolute differences of each pair of images of particular sequence they created a shape representation of the motion. The experiment demonstrated a significant increase of recognition accuracy achieved by using multimodal information. Their model improves the accuracy of state-of-the-art approaches from 82.5% reported in the literature to 91.3%, using the bi-modal face and body benchmark database (FABO) [29].

Considering all these works, one can observe that there is still a lack of comprehensive affective human analysis from body language [30] mainly because there is no clear consensus about the input and output space. The contributions of this paper are summarised as follows:

(a) We propose a different representation of affective movements, based on sequence of joints positions and orientations. Together with classification using selected neural networks and a comparison of classification performance with methods used in action recognition, for seven affective states: neutral, sadness, surprise, fear, disgust, anger and happiness.
(b) The presented algorithms utilise a sequential model of affective movement based on low level features, which are positions and orientation of joints within the skeleton provided by Kinect v2. By using such intuitive and easily interpretable representation, we created an emotional gestures recognition system independent of skeleton specifications and with minimum preprocessing requirements (eliminating features extraction from the process).
(c) Research is carried out on a new, comprehensive database that comprises a large variety of emotion expressions [31]. Although the recordings are performed by professional actors/actresses, the movements were freely portrayed not imposed by the authors. Thus, it may be treated as quasi-natural.
(d) By comparing results with action/posture recognition approaches, we have shown that emotion recognition is a more complex problem. The analysis should focus on dependencies in the sequence of frames rather than describing whole movement by general features.

This paper adopts the following outline. First, in Section 2, we describe our pipeline for automatic recognition of emotional body gestures and discuss technical aspects of each component. In Section 3, we present results obtained using proposed algorithm, which are thoroughly discussed. Finally, the paper concludes with a summary, followed by suggestions for potential future studies in Section 4.

## 2. The Proposed Method

**Figure 1:**
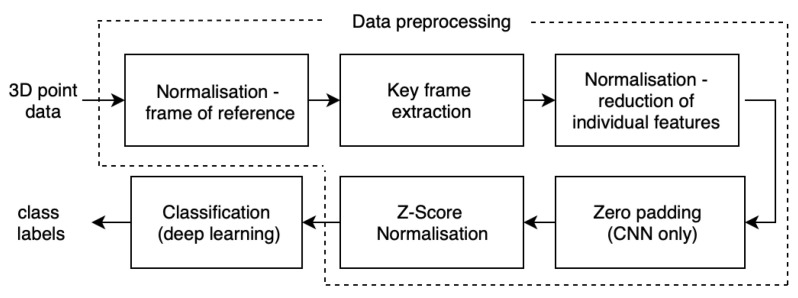
The structure of proposed emotional gestures expression recognition approach.

In this section, we present the main components of the proposed system, starting with data acquisition, followed by its pre-processing and ending with classification methods. The structure of proposed emotional gestures expression recognition approach is presented in Figure 1.

### 2.1. 3D Point Data—Emotional Gestures and Body Movements Corpora

**Figure 2:**
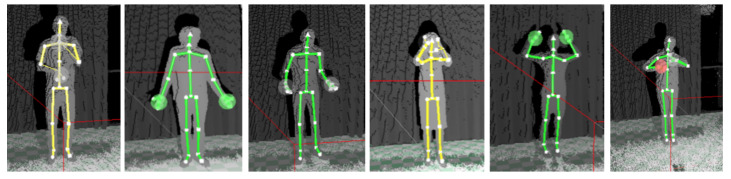
Selected frames of actor/actress’ poses in six basic emotions: fear, surprise, anger, sadness, happiness, disgust.

Motion capture data used for the purpose of this research is a subset of the multimodal database of emotional speech, video and gestures. In this work, we used our recently gathered database [31]. This section is dedicated to recordings of human skeleton. The recordings were conducted in the rehearsal room of *Teatr Nowy im. Kazimierza Dejmka w Łodzi*. Each recorded person was a professional actor/actress from the aforementioned theatre. A total of 16 people were recorded: 8 male and 8 female, aged from 25 to 64. Each person was recorded separately. Before the recording, all actors/actresses were asked to perform the emotional states in the following order: neutral, sadness, surprise, fear, disgust, anger and happiness (this set of discreet emotions was based on examination conducted by Ekman in Reference [32]). In addition, they were asked to utter a short sentence in Polish, with the same emotional state as their corresponding gesture. The sentence was *Każdy z nas odczuwa emocje na swój sposób* (English translation: *Each of us perceives emotions in a different manner*). No additional instructions were given on how a particular state should be expressed. All emotions were acted out 5 times, without any guidelines or prompts from the researchers. The total number of gathered samples amounted to 560, which includes 80 samples per each emotional state. Recordings took place in a quiet environment with no lighting issue, against a green background. Cloud point and skeletal data feeds were captured using a Kinect v2 sensor. The full body was in frame, including the legs, as shown in Figure 2. The data were gathered in the form of XEF files.

We are fully aware that there are many disadvantages of an acted emotional database. However, in order to obtain three different modalities simultaneously and gather clean and high quality samples in a controlled, undisturbed environment the decision was made to create a set of acted out emotions. This approach provides crucial fundamentals for creating a corpus with a reasonable number of recorded samples, diversity of gender and age of the actor/actress and the same verbal content. What is more, the actor/actress had complete freedom during recording: movements were not imposed and previously defined, there were no additional restrictions, every repetition is different and simulated by the actor/actress themselves. Thus, presented database may be treated as a quasi-natural one. The database is available for research upon request.

For the purpose of this research some of the samples were rejected due to technical reasons, for example, inaccurate position recognition of upper or lower extremities. The final database of affective recordings selected for this study contains 474 samples. The exact number of recordings as well as their average length for each emotional state is presented in Table 1.

**Figure 3:**
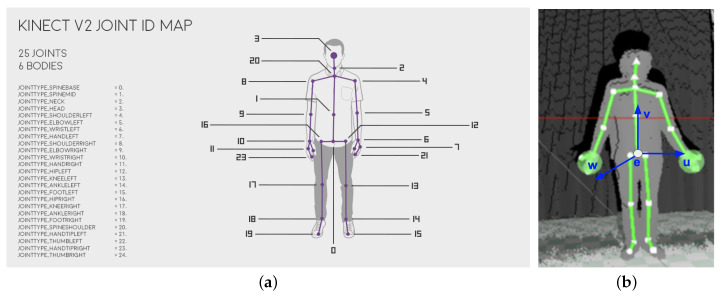
(**a**) Skeleton mapping in relation to the human body [33]. (**b**) An example frame of Kinect recording showing the skeleton.

Data acquired from the Kinect v2 determines the 3D position and orientation of 25 individual joints, as shown in Figure 3a. The position of each joint is defined by the vector [x,y,z], where the basic unit is 1m and the origin of the coordinate system is Kinect v2 sensor itself. The orientation is also determined with three values expressed in degrees. The device does not return orientation values of head, hands, knees and feet.

### 2.2. Preprocessing

Raw Kinect v2 data output needs to be subjected to several steps of processing before it can be used in classification—each step is described in following section. The assumption of this research was to reduce data preprocessing to minimum in order to make the path between data acquisition and classification as short as possible, maintaining effective emotion recognition at the same time.

#### 2.2.1. Normalisation—Frame of Reference

Kinect v2 provides data of 3D joints position and orientation, in the space relative to the sensor itself [x,y,z] (where *x* is pointing left from the sensor, *y* is pointing upwards, *z* is the forward axis of the sensor). This kind of data is influenced by the distance between the actor/actress and the sensor during recording. Thus, skeleton coordinates had to be projected from the sensor space [x,y,z] onto a local space of the body [u,v,w] with the center of this space in the SpineBase joint of the Kinect skeleton (presented in Figure 3a, called the main joint or root joint), where *u* is pointing left, *v* is pointing up, *w* is pointing forward in relation to the SpineBase joint, all [u,v,w] coordinates were calculated in respect to the main joint rotation, as shown in Figure 3b. As a result, a vector containing the positions and orientation of all joints in relation to the main one was obtained. This operation is performed for each frame in every sample. Positions and orientations of the main joint in the first frame are treated as the initial state, while the changes in the displacement or rotation of the main joint in subsequent frames are calculated in relation to the first frame.

#### 2.2.2. Key Frame Extraction

**Figure 4:**
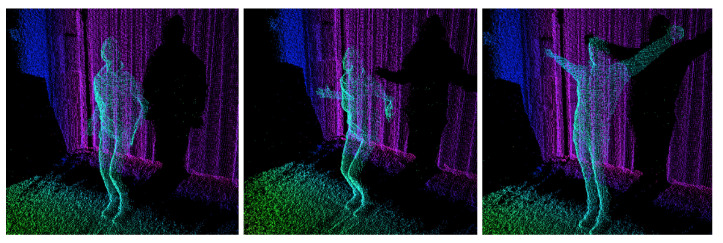
Sequence of three key frames extracted from point cloud data representing happiness.

Gestures and body movements can be analyzed as a set of key frames. The key frame should contain crucial information about a particular pose for a given motion sequence. For this purpose, body movement should be divided into separate frames as can be seen in Figure 4.

There are many methods for key frame extraction. Most of them fall into three categories, namely, curve simplification (CS), clustering and matrix factorisation [34]. For the purpose of this research, CS method was used. In this method, the motion sequence is represented as a trajectory curve in 3D space of features and CS algorithms are applied to these trajectory curves. CS utilises Lowe’s algorithm [35] for curve simplification, which represents the values of a single joint in a sequence of motion. Starting with the line connecting the beginning and the end of the trajectory, the algorithm divides it into two sublines (intervals), if the maximum deviation of any point on the curve is greater than a certain level of error. The algorithm performs the same process recursively for each subline, until the error rate is small enough for each subline. In this study, we examined the following values of error rate: 1 cm, 2 cm, 3 cm, 5 cm, 10 cm and 15 cm. For the error rate of 1 cm and 2 cm, the obtained number of key frames is almost identical to the number of frames of the recording, even for neutral state in which the actor/actress stay almost still. Thus, this level of error rate is considered as a Kinect v2 measurement error (especially in the case of hand movement, which is described in Section 2.3). For the error rate of 10 cm and 15 cm, the obtained number of key frames is not sufficient to adequately describe emotional movement. The average number of key frames oscillates around 2, which means that only a few frames between the first and the last one were selected. Thus, error rate values of 1 cm, 2 cm, 10 cm and 15 cm were excluded from further analysis.

#### 2.2.3. Normalisation—Reduction of Individual Features

It is assumed that every human is built in proportion to his or her height and the length of legs and arms is proportional to the overall body structure [36]. To unify the value of the position of the joints between the higher and lower individuals, we propose normalisation based on the distance between two joints with the lowest noise value of their position on all recordings: SpineBase and SpineShoulder. The distance used for normalisation is measured for each frame of the actor/actress’s neutral recordings. Normalisation of all joints within a given sequence of frames follows Equation (1), where skeleton consists of 25 joints, $d_{i}$ is the distance vector between the *i* and $J_{0}$ joints normalised to the median of distances between the joints $J_{0}$ and $J_{20}$ (SpineBase and SpineShoulder) of all neutral recordings for each individual.

(1) $$d_{i}=\frac{J_{i}}{\tilde{J_{20}-J_{0}}}$$

where i=1,…,25 is the number of joints. This process is performed for all joints, relative to the skeleton in the neutral position of particular individual. Neutral state is used to preserve information about special movements such as jump or squat occurring in emotional recordings (e.g., joyful hop). Considering the same degree of freedom of each body part for all recorded individuals, values of joint orientation did not require any additional processing.

The output of the key frames extraction is a set of sequences of varying lengths, which can not be considered as an input for all types of classifiers, in our case CNN. In order to unify the length of the sequences, we applied zero padding algorithm to prepare the data for CNN.

Next, all sequences are subjected to z-score normalisation, which is a widely used step to accelerate the process of neural networks learning [37,38,39]. For the purpose of this research we apply *sequence-wise normalisation* [38] for each key frame sequence. In this method, mean and standard deviation is calculated among data from all sequences excluding zero frames added during the previous step.

### 2.3. Datasets Division

During data preparation, a relative average quantity of motion (distance covered by a specific joint) was measured for each emotional state. Calculations were made according to the formula (2).

(2) $$avg_{je}=\sum_{ne=1}^{N_{e}}\frac{|p_{je}\left(f_{ne}\right)-p_{je}\left(f_{ne}-1\right)|}{F_{ne}N_{e}}$$

where: j=0,…,25—the number of the joint; *e*—emotional state (Ne—neutral, Sa—sadness, Su—surprise, Fe—fear, An—anger, Di—disgust, Ha—happiness); $N_{e}$—is a number of recordings per emotion *e*; ne = 1,...,$N_{e}$—the index of the emotional state *e* recording; $F_{ne}$—is a number of frames per recording *n* of emotional state *e*; $f_{ne}$ = 2,...,$F_{ne}$—frame index in recording *n* of emotional state *e* (excluding first frame); $p_{je}\left(f_{ne}\right)$—position of joint *j* in frame $f_{ne}$ in hierarchical local coordinates.

**Figure 5:**
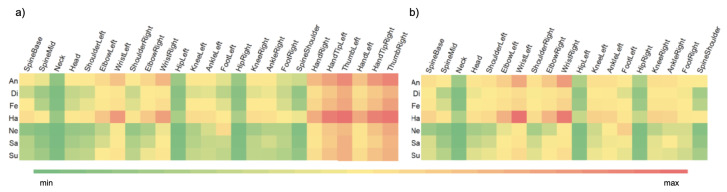
Heat-map presenting distribution of joints involvement for particular emotional state (**a**) for all joints (**b**) excluding hands.

For each joint, the calculated values are relative, based on changes in the local coordinate system of the given joint, the centre of which is located in a superior joint in hierarchical skeleton construction (e.g., for the WristRight joint corresponding to the position of right hand wrist, the origin of the local coordinate system is the ElbowRight joint corresponding to the position of the right hand elbow. These calculations were made separately for each emotion. The results are shown in Figure 5.

One can observe in Figure 5a that the largest involvement in emotional expression is observed for hands and thumbs (HandTipLeft, HandTipRight, HandTipLeft, HandTipRight, HandLeft, HandRight). However, the intensity of movement of these particular joints is caused by the measurement error of Kinect v2. Thus, in further analysis it is assumed that the hand position is determined by position of the wrists (WristLeft i WristRight) and all hand related joints were excluded from the datasets. According to Figure 5b, the largest involvement is observed for wrists and arm related joints, which is common for emotion expression. It is worth emphasising that the involvement of legs is visible, especially for the knees (KneeLeft, KneeRight) and ankles (AnkleLeft, AnkleRight).

Most state-of-the-art research focuses only on the upper body, thus in this study, the influence of leg movement on affective gestures was examined. In addition, we investigated which type of data (joint orientation, position or mixture of both) is best suited for the classification of emotional sates from gestures. In order to conduct such research we examined the datasets presented in Table 2.

### 2.4. Classification—Models of Neural Networks

The final step of the proposed method is classification, which aims to assign input data to a specific category *k* (in this case: neutral state, sadness, surprise, fear, anger, disgust, happiness). In this work, we apply different deep learning Neural Networks (NN) to the proposed combination of datasets Table 2 in order to compare their performances, based on the recognition rates. We use a Convolutional Neural Network (CNN), a Recurrent Neural Network (RNN) and a Recurrent Neural Network with Long Short-Term Memory Network (RNN-LSTM) with low level features (positions and orientation of joints within the skeleton), in terms of motion emotion recognition efficiency. The proposed approach of adjusting the abovementioned neural networks to motion sequence analysis is presented in the following section.

#### 2.4.1. Convolutional Neural Network

**Figure 6:**
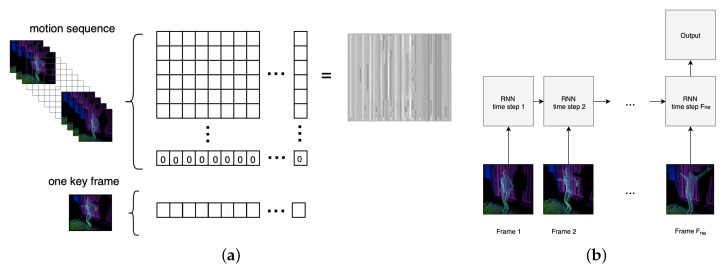
(**a**) The process of using a Convolutional Neural Network (CNN) for gestures-based emotion recognition shows the process of creating an matrices based on motion sequence. (**b**) The process of using a Recurrent Neural Network (RNN) for motion sequence analysis—each time step of the motion sequence is evaluated by a RNN.

The scope of use of CNNs has expanded greatly to different application domains, including the classification of signals representing emotional states [40,41]. Due to its well configured structures consisting of multiple layers, this kind of network is able to determine the most distinctive features based on enormous collections of data. The possibility of reducing the number of parameters required for images over a regular network makes CNN the most commonly used classifier for image processing. CNN considers an image as a matrix and uses the convolution operation [42] to implement a filter, which is sliding through the input matrix. In a multi-layered CNN, the input of each convolution layer is comprised of the filtered output matrix of the previous layer. The convolutional filter values are adjusted during the training phase. The process of using a CNN for gestures-based emotion recognition from sequence of movement is presented in Figure 6a.

#### 2.4.2. Recurrent Neural Network

RNNs allow operation directly on time sequences. They are successfully applied to tasks involving temporal data such as speech recognition, language modelling, translation, image captioning or gestures analysis. In RNN, the output of the previous sequence time step is taken into consideration when calculating the result of the next one. However, standard RNN does not handle long term dependencies well, due to the vanishing gradient problem [43].

The Long Short Term Memory network (RNN-LSTM) is an extension for RNN, which works much better than the standard version. In RNN-LSTM architecture, RNN uses gateway units in addition to the common activation function, which extend its memory [44]. Such an architecture allows the network to learn and “remember” dependencies over more time steps, linking causes and effects remotely [45]. The process of using a RNN and RNN-LSTM for gestures-based emotion recognition sequence of movement is presented in Figure 6b.

## 3. Results and Discussion

### Selection of the Optimal Classification Model

For each of the neural network types mentioned in Section 2.4, the following architectures were tested:

- CNN networks containing from 2 to 3 convolution layers (each convolution layer was followed by a max pooling layer) followed by 1 to 2 dense layers, from 50 to 400 neurons for convolution and 50 to 200 for dense neurons;
- RNN networks containing from 2 to 4 layers, built from 50 to 400 neurons;
- RNN-LSTM networks containing from 2 to 4 layers, built from 50 to 400 neurons;

For all NN types, separate models were built increasing the neuron count on each layer by 25 for each new model (i.e., for RNN starting with a network containing 2 layers of 50 recurrent neurons and finishing with 4 layers containing 400 neurons). Table 3 shows the results obtained using three types of neural network for the above mentioned datasets. For CNN, the best results were obtained for a network of 4 layers, 3 layers of convolution neurons 250, 250, 100 for each layer respectively and a dense layer of 100 neurons. For RNN best results were obtained for a 3 layer model with 3 recurrent layers of 300, 150, and 100 neurons. RNN-LSTM achieved best results for a 3 layer architecture of 250, 300, 300 neurons. In addition, all NNs had a single dense layer of 7 neurons as the output layer. We used 10-fold leave-one-subject-out cross-validation and repeat the process for 10 iterations, averaging the final score. All NNs were trained using ADAM [46] for gradient descent optimisation and cross–entropy as the cost function, as it is a robust method based on well known classical probabilistic and statistical principles and is therefore problem-independent. It is capable of efficiently solving difficult deterministic and stochastic optimisation problems [47]. Training was set to 500 epochs with an early stop condition if no loss decrease was detected for more than 30 epochs.

One can easily observe that the best results (69%) were obtained using RNN-LSTM on the *P* set containing position of all skeletal joins (upper and lower body). In general, this set of features gives the best results for all types of networks (58.1% for CNN, 59.4% for RNN). This suggests that this kind of features provide the best description for emotional expressions from all considered feature types. In case of the *PU* set, results for all networks are lower than 5%, which indicates the effect of the lower part of the body on recognition. Using orientation *O* as a features set, even if complimenting the position (*PO* or *POU*), results in much lower recognition. According to Table 3 results indicate a slight impact of error rate—better results were achieved using the 3 error rate almost for every dataset and NN, in few cases the results were equal. This may suggest that even a small movement or displacement can affect the recognition of emotions and the error rate of 5 cm might not be precise enough to represent all relevant movement data.

In addition, the experiment was conducted on sequences without the keyframing step in the pre-processing (containing all the recorded frames) for all NN models and all the datasets. The results of classification were 5–10% lower (depending on the model and set) than those acquired by key frames with error rate of 3 cm. Moreover, the time of NN training rose significantly due to a large increase in the data volume. Lower recognition results for sets without keyframing might have been caused by the Kinect v2 sensor noise, as the device output is not very precise and produces small variations in returned positions and orientations from frame to frame. This can be mitigated by applying filtering on the signal, however it is a time and computational consuming process, which does not fall into the assumption of reducing data pre-processing to a minimum. In our approach, the keyframing process allowed us to avoid the sensor precision related issues.

Performance of the proposed NN models was compared with the state-of-the-art NN architecture, ResNet. It has won several classification competitions, achieving promising results on tasks related to detection, localisation and segmentation [48]. The core idea of this model is to use a so-called identity shortcut connection to jump over one or more layers [49]. ResNets use the convolutional layers with 3×3 filters, which are followed by batch normalisation and rectified linear unit ReLU. Plenty of experiments showed that the use of the shortcut connections makes the model more accurate and faster compared to their equivalent models. We recreated the exact process as described in Reference [48], as the results obtained for action recognition in Reference [48] look very promising (accuracy over 99%) and as initially assumed, the method might be applicable for emotional gestures classification. The 3D coordinates of the Kinect skeleton (from our *P* and *PU* datasets) were transformed into RGB images. The sets were also augmented according to the description in the source paper. For our experiment, we prepared the testing and training set following the 10-fold leave-one-subject-out cross-validation method, meaning that the testing set did not contain the training samples and samples obtained from training set samples augmentation. Accuracy achieved using ResNet is significantly lower than that of the other NN types. This might be caused by the size of the original dataset, which contains only 474 unique samples and the process of argumentation presented in Reference [48] does not produce a diverse enough set to train such a deep NN.

**Figure 7:**
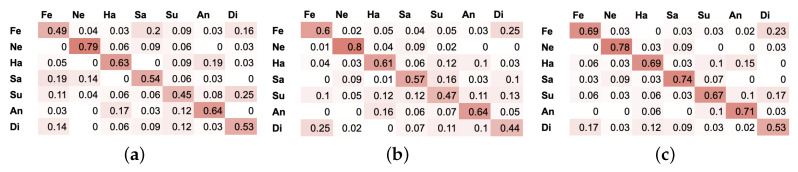
Confusion matrix for (**a**) CNN on *P* set with 3 cm error rate (**b**) RNN on *P* set with 3 cm error rate (**c**) RNN-LSTM on *P* set with 3 cm error rate. Seven emotional states: Ne—neutral, Sa—sadness, Su—surprise, Fe—fear, An—anger, Di—disgust, Ha—happiness.

For each type of neural network, the best results are presented in a form of confusion matrix (see Figure 7). One can observe that the best results were obtained for the neutral state as it differs greatly from other expressions (the actor/actress stood still, while there was a relatively bigger amount of movement while expressing other states).

Happiness, sadness and anger have a high rate of recognition and are sporadically classified as other emotions, as gestures in those three states are highly distinctive and differ from other emotional states (in terms of dynamics, body and limb positions and movement), even when the gestures are not exaggerated. Disgust and fear were confused with one another most frequently, this might be caused by the way they were performed by the actor/actress, as this confusion pattern is analogous for all three NN types. It is clearly visible on the recordings that those two emotions were acted out very similarly in terms of gestures (usually backing out movement with hands placed near head or neck for both states).

**Figure 8:**
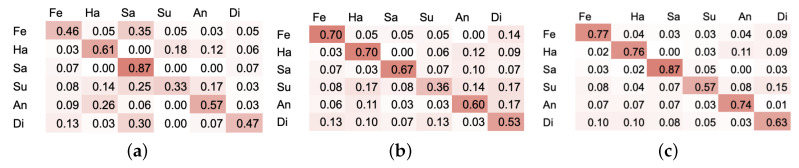
Confusion matrices for (**a**) CNN on *P* set with 3 cm error rate (**b**) RNN on *P* set with 3 cm error rate (**c**) RNN-LSTM on *P* set with 3 cm error rate. Six emotional states: Fe—fear, Ha—happiness, Sa—sadness, Su—surprise, An—anger, Di—disgust.

**Figure 9:**
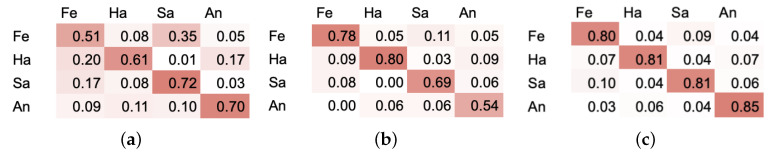
Confusion matrices for (**a**) CNN on *P* set with 3 cm error rate (**b**) RNN on *P* set with 3 cm error rate (**c**) RNN-LSTM on *P* set with 3 cm error rate. Four emotional states: Fe—fear, Ha—happiness, Sa—sadness, An—anger.

Since the recognition accuracy of the neutral class far exceeds other emotional states, as the samples for this state contain the least amount of motion and it differs from all the other states greatly, in the next step we analyse two sets without this class. From the first one we merely exclude neutral state, thus it consists of sadness, surprise, fear, anger, disgust and happiness. The second set contains emotional states, which are most commonly used in the literature: sadness, fear, anger, and happiness. Experimental results of the above-mentioned datasets are presented in Table 4. As in the case of seven classes, the best results were obtained using *P* set. Similarly, RNN-LSTM proved to be the most effective, providing 72% in case of 6 classes and 82.7% in the case of 4. Confusion matrices for the above-mentioedn sets are presented in Figure 8 and Figure 9.

In order to compare the proposed method with other classification methods, we calculated the most commonly used features, such as kinematic related features (velocity, acceleration, kinetic energy), spatial extent related features (bounding box volume, contraction index, density), smoothness related features, leaning related features and distance related features. During features extraction we strictly followed approach presented in Reference [23], since the authors obtained very promising results on a database derived from Kinect recordings. We juxtaposed several well known classification methods to verify the above-mentioned features and their effectiveness in gestures-based emotion recognition. The obtained results are presented in Table 5.

To determine the performance of the above-mentioned classifiers we used the WEKA [50] environment. All parameters of the classifiers were set empirically in order to achieve the highest efficiency. As one can easily observe, the best results were obtained in the case of Random Forests. However, it should be emphasised that none of the methods listed above achieve better results than the proposed approach. This is a result of the generalisation of features from the whole recording, an approach which might be appropriate for simple gestures recognition; however, it becomes inaccurate for more complex and non-repeatable expressions.

## 4. Conclusions

In this paper, we presented a sequential model of affective movement as well as how different sets of low level features (positions and orientation of joints) performed on CNN, RNN and RNN-LSTM. The training and testing data contained samples representing seven basic emotions. The database consisted of recordings of constant affective movements, in contrast with other research, which is mostly reduced to specific single gesture recognition. Thus, we did not analyse solely separated selected frames but the whole movement as a unit. This experiment highlighted how challenging the task of recognising an emotional state based merely on gestures might be. The performance was much lower than in the case of particular gesture recognition; however, it was still higher than a human’s performance (63%) [31].

The obtained results showed that body movements can serve as an additional source of information in a more comprehensive study. Thus, for future work we plan to combine all the three modalities, namely audio, facial expressions and gestures, which are signals perceived by a healthy human during a typical conversation. We believe that additional patterns extracted from affective movement may have a significant impact on the quality of recognition, especially in the case of emotion recognition in the wild [41]. In addition, we plan to extend our analysis using the Denspose [51] method and fuse and juxtapose with features provided by Kinect v2.

What is more, we will explore and compare methods used for action recognition, such as those presented in References [52,53,54], as they provide interesting expansion of the models used in this paper. For example, in Reference [52] the authors use a similar RNN-LSTM network architecture, instead of raw skeletal data, geometrical features extracted from the skeleton are fed to the NN. Also an interesting approach for RNN-LSTM is presented in Reference [53], where spatial attention joint-selection gates and temporal attention with frame-selection gates are added to RNN-LSTM. In Reference [54], the authors used F2C CNN -based network architecture for action recognition, with superior results compared to other classification modes. We plan to incorporate methods used in action recognition for the purpose of gesture based emotion classification, as the problem poses similar challenges in both areas.

## Figures and Tables

**Table 1:** The amount of samples used in the research and the average length of recordings per emotion (in seconds).

Emotional State	Neutral	Sadness	Surprise	Fear	Anger	Disgust	Happiness
No. of samples	64	63	70	72	70	65	70
Average recordings length in second	3.7	4.16	4.59	3.79	4.15	4.76	4.03

**Table 2:** Input datasets for classification

Dataset	Dataset Content	Dataset Features Count
PO	Positions and orientation, upper and lower body	115
POU	Positions and orientation, upper body	67
P	Positions, upper and lower body	58
O	Orientation, upper and lower body	58
PU	Positions, upper body	34
OU	Orientation, upper body	34

**Table 3:** Classification performances of different feature representations in for the set of 7 basic emotions. Numbers in bold highlight the maximum classification rates achieved in each column. PO—Positions and orientation, upper and lower body, POU—Positions and orientation, upper body, P—Positions, upper and lower body, O—Orientation, upper and lower body, PU—Positions, upper body, OU—Orientation, upper body.

Features Set	PO		POU		P		O		PU		OU	
Error Rate	3	5	3	5	3	5	3	5	3	5	3	5
CNN	56.6	54.8	38.8	38.8	**58.1**	56.8	33.6	33.0	41.6	38	50.2	49.0
RNN	55.4	55.2	49.2	49.0	**59.4**	59.4	36.4	33.8	54.6	54.2	34.4	31.8
RNN-LSTM	65.2	59.6	64.6	61.25	**69.0**	67.0	55.0	54.6	65.8	64.2	54.2	53.8
ResNet20	-	-	-	-	27.8	27.5	-	-	25	23.7	-	-

**Table 4:** Classification performances of different feature representations for the set of basic emotions. PO—Positions and orientation, upper and lower body, POU—Positions and orientation, upper body, P—Positions, upper and lower body, O—Orientation, upper and lower body, PU—Positions, upper body, OU—Orientation, upper body.

Features Set	PO		POU		P		O		PU		OU	
#Emotions / #Classes	6	4	6	4	6	4	6	4	6	4	6	4
CNN	50.5	55.2	51.5	55.5	**54.2**	**63.6**	47.8	50.5	53.7	60.7	47.4	49.2
RNN	54.4	66.8	58.6	70.8	**59.2**	**80.8**	39	55.2	54.4	66.8	40	57.2
RNN-LSTM	66.2	80	59.6	74.2	**72**	**82.7**	51.8	62.4	64.6	75.8	47.4	58.9
ResNet20	-	-	-	-	30.6	40.2	-	-	30.1	39.7	-	-

**Table 5:** The performance of some well-known classifiers.

Classifier	#Emotions/#Classes		
	7	6	4
J48	45.36	37.07	56.36
Random Forests	**52.95**	**50.73**	**64**
k-NN	43.46	42.92	61.09
S-PCA + k-NN	35.86	37.33	51.27
SVM	41.98	42.93	59.27
MLP	42.19	46.09	61.45

## Abbreviations

The following abbreviations are used in this manuscript:

ADAM	Adaptive Moment Estimation
CNN	Convolutional Neural Network
CS	Curve Simplification
HMM	Hidden Markov Model
k-NN	k-nearest neighbors
LSTM	Long short-term memory
NN	Neural Network
RBM	Restricted Boltzmann Machine
RNN	Recurrent Neural Network
ResNet	Residual Network
SVM	Support Vector Machine
MLP	Multilayer perceptron
